# Exercise interventions for stress reduction in older adult populations: a systematic review of randomized controlled trials

**DOI:** 10.1080/21642850.2022.2125874

**Published:** 2022-09-25

**Authors:** Ryan Churchill, Kelly Teo, Lucy Kervin, Indira Riadi, Theodore D. Cosco

**Affiliations:** a Department of Gerontology, Simon Fraser University, Vancouver, Canada; b Oxford Institute of Population Ageing, University of Oxford, Oxford, UK; c Harper, The Bloomsbury Building, London, UK

**Keywords:** Stress, exercise, qigong, mind–body, older adults

## Abstract

**Background and Objectives:**

To assess which forms of supervised exercise are effective in reducing psychological stress in older adults.

**Research Design:**

Systematic Review.

**Methods:**

Four electronic databases (PubMed, Web of Science, PsycInfo, and SportDiscus) were searched in February of 2021. Randomised controlled trials (RCTs) investigating supervised exercise interventions for psychological stress reduction in adults aged 50 + were included in this review. Data on type, intensity, and duration of the intervention were also extracted.

**Results:**

854 studies were identified by the search strategy. Twelve RCTs met inclusion criteria. Trials involving low-intensity qigong and trials combining aerobic and anaerobic or aerobic and nutrition/diet education demonstrated the strongest evidence for stress reduction.

**Discussion and Implications:**

Exercise may reduce stress in older adults. Suitable duration of programme ranges from 3 months to 1 year. Light to moderate activity is recommended for best results, with qigong being the most consistent and common exercise.

## 1. Introduction

Stress is the body’s reaction to a threatening stimulus resulting in various affective, physiological, biochemical, and/or cognitive–behavioural manifestations, threatening the body’s natural homeostasis. These may include. high blood pressure, increased heart rate and/or muscle tension (Chrousos & Gold, 1992). Stress differs from other psychological phenomena, such as anxiety, which is characterised by persistent excessive worries that occur without a distinguishable or tangible stressor (APA, 2022a), or depression, which is characterised by anhedonia, significant weight loss/gain, sleep issues, loss of concentration, and/or feelings of worthlessness (APA, 2022b). Psychological stress has been shown to have wide-ranging negative effects on adults’ overall health (Nielsen et al., 2008). As people age, challenges may arise, exacerbating stress levels, such as increased loneliness, bereavement, increased frailty, and/or transitioning out of the workforce (Scott et al., 2011). Due to increasing longevity worldwide, a higher proportion of adults are reaching older ages, thus increasing the proportion of individuals encountering age-related stressors (Marengoni et al., 2011). Left unaddressed, the possibility of numerous negative stress-related outcomes may develop. Increased stress in older adults has been linked to depression, cognitive impairment, and declines in overall health status (Juster et al., 2010).

Exercise presents a potential mechanism for alleviating stress safely and cheaply (Gerber et al., 2016). The Centers for Disease Control and Prevention (CDC, 2017) in their glossary of terms, define exercise as ‘a type of physical activity, exercise is planned, structured, repetitive, and purposive, in the improvement or maintenance of one or more components of physical fitness. It is the objective, general, or structured movement of the body, increasing energy expenditure.’ For the purpose of this review (both in theory and inclusion criteria), exercise will be the predominant type of physical activity discussed.

Previous reviews related to older adults’ mental health and physical activity primarily focused on depression (Forsman et al., 2011) and anxiety (Mochcovitch et al., 2016) or specific exercises such as Tai Chi (Wayne et al., 2014). Also, reviews including stress as a primary outcome usually involved younger cohorts (Gerber & Pühse, 2009). One review that investigated stress-buffering influence of exercise found over half the studies showed evidence of this effect, (Gerber & Pühse, 2009) though they remark older adult-focused research is scarce and more is necessary to make concrete assertions. One primary study did find physical activity provided some protective buffers for increased stress in old age (Unger et al., 1997).

To our knowledge, this is the first systematic review examining psychological stress as the primary outcome of measurement following a supervised exercise intervention within adult populations over the age of 50. This review aims to articulate best evidence for duration, intensity, and type of exercise in stress reduction for older adults so they may navigate stressful circumstances as effectively as possible.

## 2. Methods

This systematic review is registered with PROSPERO at ‘blinded for review’. More detail regarding methods employed in this review can be found in the protocol ‘blinded for review’.

### 2.1. Search strategy and selection criteria

This systematic review followed guidelines outlined in the Preferred Reporting Items for Systematic Reviews and Meta-Analyses (PRISMA) checklist (Moher et al., 2015). A search was conducted within four electronic databases: PubMed, Web of Science, PsycInfo, and SportDiscus. The searches were independently completed by two reviewers. The searches combined terms related to psychological stress, exercise, and older adults. Inclusion and exclusion criteria can be found in Table 1.

**Table 1.** Inclusion/Exclusion criteria.

Inclusion criteria	Exclusion criteria
Age ≥ 50	Self-reported levels of exercise
Pre + post intervention stress score	Non-human
Randomised Controlled Trials trials	Severe cognitive impairment
Psychological report of stress	Protocols, reviews, editorials
Peer-reviewed articles	
Supervised objective report of exercise	
Primary data analysis	

### 2.2. Studies

This systematic review examines randomised controlled trials that investigate psychological stress levels before and after an exercise intervention. The studies included were original and peer-reviewed. No limitations on publication date, language, or country of origin were imposed.

### 2.3. Participants

Studies including older adults without severe cognitive impairment that may make exercise hazardous were included. Included studies had to include adults 50 years of age or older. If they included multiple age group stratification, studies had to detail data relevant to the research question and specific to adults aged 50 + . Various different trials of older adults were included (those with cardiovascular disease, cancer, healthy but inactive), but for the scope of this review, they were not compared as it relates to how they dealt with stress post exercise.

### 2.4. Interventions

This review included both aerobic and anaerobic interventions and both individual and group-based exercise. There was no limitation on type of exercise. Exercise interventions had to be supervised and objectively measured. Any interventions with only self-reported exercise were excluded. This was done to reduce bias with regards to self-reporting activity levels. Interventions could vary in intensity.

### 2.5. Comparators

The treatment in the comparator arm had to be less intensive than the treatment in the intervention. Examples include: usual care, exercise education, programme waitlist or non-exercise.

### 2.6. Outcomes

The main outcome of interest was perceived psychological stress, defined as, ‘a particular relationship between a person and the environment that is appraised by the person as taxing or exceeding his or her resources and endangering his or her well-being’ (Lazarus & Folkman, 1984, p. 19), Distress was also included – defined as either severe or chronic stress or both. While stress is a normal reaction to an event, distress can be considered extreme and negative. The stress score was assessed using various psychological stress questionnaires or assessment tools. While the Perceived Stress Scale (PSS) is the most widely used psychological instrument for assessing perceived stress, (Cohen et al., 1983) other scales evaluating psychological stress were accepted. All included studies needed a baseline score and a post intervention score.

### 2.7. Search methods

Aside from SPORTDiscus (all fields were searched), these search terms were examined using ‘title and/or abstract’ searching to ensure specificity.

Articles were imported into Endnote. Duplicates were removed. Double independent screening was conducted by co-authors for titles and abstracts followed by full text screening.

### 2.8. Data extraction

Data extracted included: Title, authors, year of publication, country of publication, demographic characteristics, sample size, intervention and control type and dosage, delivery setting, analysis type, stress score, duration of trial, BMI, smoking rate, and dropouts. Some of these can be found in abbreviated form in Table 2.

**Table 2.** Study demographics.

Author(s)	Year	Country	Population	Age	n	Dropout
Campo et al.	(2014)	U.S.A.	prostate cancer survivors, sedentary	55+	29	11/27.5%
Cormie et al.	(2015)	Australia	men with prostate cancer, 46% past smoker, two current smokers	stratified ≥70, ≤ 70, intervention mean 70	63	8/12.7%
Courneya et al.	(2017)	Canada	post-menopausal women at risk for breast cancer, inactive, non-smoker, BMI 22-40, v02 < 34	50–74	400	14 / 3.5%
Ehlers et al.	(2017)	U.S.A.	community-dwelling, read and write in English, right-handed, low active/inactive, MMSE >23, cognitive status > 21	60–79	247	39/15.9%
Gothe et al.	(2016)	U.S.A.	Low-active, healthy community dwelling	55–79	108	10 / 9.3%
Imayama et al.	(2011)	U.S.A.	overweight/obese, post-menopausal women, BMI > 25, low active, no breast cancer, no hormone replacement past 3 months, non-smoking	50–75	439	57 / 13%
King et al.	(1993)	U.S.A.	no cardiovascular disease/stroke, no medication hypertension/hyperlipidemia, women: no hormone replacement therapy 20% smokers	50–65	357	NA
Pourhabib et al.	(2018)	Iran	heart failure	60–74	53	7 / 11.7%
Puterman et al.	(2018)	U.S.A.	family caregivers of relatives with dementia, non-smokers	50–75	68	4 / 5.9%
Vaapio et al.	(2007)	Finland	fallen once in past year, living at home	65+	513	61 / 10.3%
Xiao et al.	(2018)	China	cardiovascular disease, 19.4% smoking	≤64, ≥65 (65%)	129	5 / 3.7%
Zhang et al.	(2016)	China	chronic COPD	58–72	130	18 / 12.2%

BMI: Body Mass Index, Vo2: Volume of Oxygen, COPD: Chronic Obstructive Pulmonary Disease, MMSE: Mini-mental state exam.

### 2.9. Quality assessment

To assess the quality of trials in this review, Cochrane Risk of Bias Tool Version 2 (ROB-2) was used. The ROB-2 tool was used in this review as it has recently become the standard in assessing risk of bias of randomised controlled trials (Jørgensen et al., 2016). It evaluates the following: (a) random sequence generation, (b) allocation concealment, (c) selective reporting, (d) incomplete outcome data, (e) performance bias (blinded participants and/or personnel), and (f) detection bias (blinded outcome assessment). Each trial was given a subjective grade of low, high, or unclear (Sterne et al., 2019). Low, high, and unclear risks of bias are displayed in green, red, and yellow, respectively (Table 3). Studies were denoted as having unclear risk of bias if insufficient information was provided or information was unclear, for minimum one of six categories.

**Table 3.** Risk of bias in included studies.

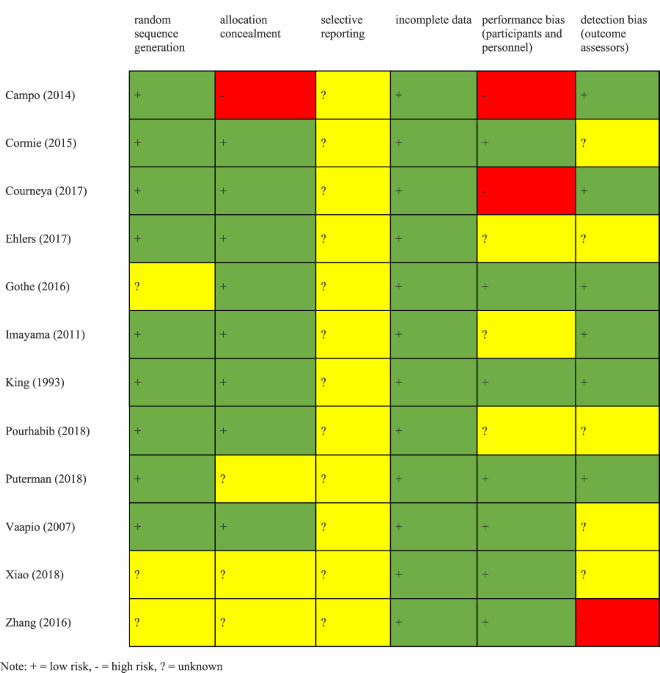						

### 2.10. Narrative synthesis

In the instance that methodological heterogeneity would prevent meta-analyses, a narrative synthesis of the literature would be conducted instead. A narrative synthesis is used in systematic reviews to synthesise findings from multiple studies that use primarily text to summarise and explain findings of the review. A framework developed for narrative syntheses (Popay et al., 2006) suggests systematic reviews without a meta-analysis usually consist of theory of why or why not an intervention works and for whom, developing a synthesis of findings from included studies, exploring relationships in the data, and assessing robustness of the synthesis.

## 3. Ethics statement

As this paper is a review, no ethics application was necessary.

## 4. Results

### 4.1. Included studies

**Figure 1.**
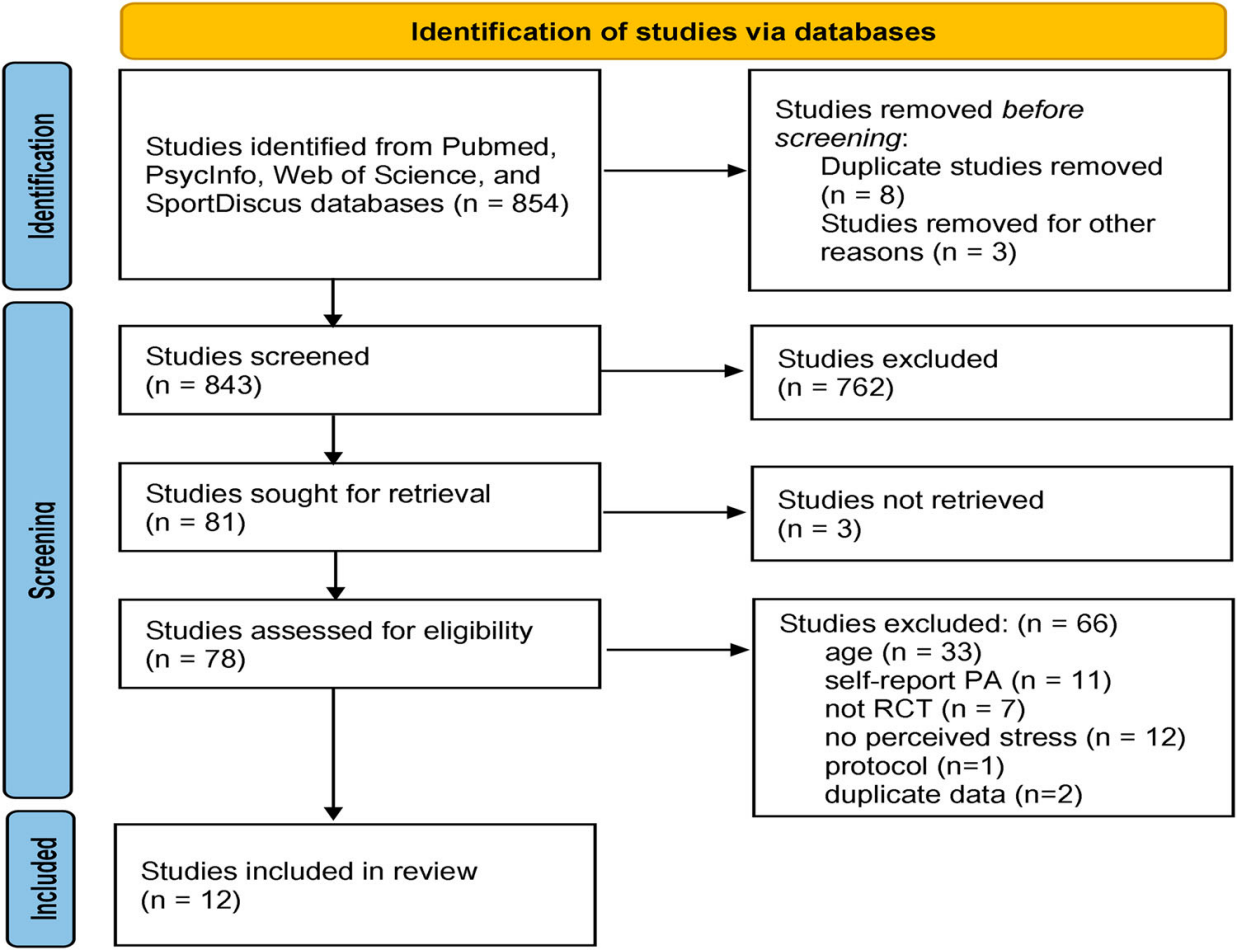
PRISMA flowchart: Screening process for study inclusion.

The databases searched yielded 854 articles after removal of duplicates. After screening titles/abstracts, 81 articles remained. Of these 81 studies, three were unavailable through the institutional library accessible to the reviewers. Attempts were made to contact lead authors (via ResearchGate) to obtain access to full texts; however, no response was received, leaving 12 articles for inclusion (Figure 1).

### 4.2. Study characteristics

#### 4.2.1. Key findings

Findings from all articles are presented below (Table 4). Information extracted includes: exercise type, trial length, exercise intensity, intensity defined, exercise duration, and findings related to stress. Following will be detailed narrative summaries of each exercise.

**Table 4.** Exercise interventions and findings.

Author	Exercise	Trial length	Intensity rank	Intensity defined	Control	Duration	Stress measure	Findings
Campo et al.	Qigong	2x week: 3 months	moderate	BORG scale (perceived exertion): median = 4.3, range = 1.8-8.4	stretching	1 h	Distress (BSI-18)	Qigong associated with sig. decrease in distress vs. control (*p* < 0.05)
Xiao et al.	Baduanjin (Fitness Qigong)	5x week: 4 months	Light	Not applicable	Usual activities	24min	Emotional distress SEMCD-6	Qigong group sig. higher confidence to manage emotional distress (*p* = 0.011)
Zhang et al.	Qigong Yijin Jing	7x week: 6 months	Light	Heart rate increase < 20 bpm, Breathing rate increase <5 times/min,	Usual care	1 h	Distress (RESE questionnaire)	Qigong group sig. more capable managing distress vs. control + self-management exercise groups (*p* < 0.001); effect increases 1-3-6 months
Gothe et al.	Hatha Yoga	3x week: 2 months	Light	Beginner class increase complexity over trial	Stretching	1 h	PSS	No group difference, both sig. decrease stress pre-post (*p* = 0.001)
Ehlers et al.	Dancing, walking, walking + nutrition	3xweek: 6 months	light/moderate	Dancing: increase in intensity, walking: 50-60%MHR 60-75%	Strength/stretching/stability	1 h	PSS	perceived stress decreased in both control and intervention (*p* < 0.02) not between exercise mode (*p* > 0.29) or exercise vs. control (*p* > 0.11)
Courneya et al.	Aerobic Choice	5x week: 1 year	Light/moderate	65-75% MHR	Minimum recommended exercise (150 min/week)	1 h	PSS	NS (*p* = 0.69)
Puterman et al.	Aerobic Choice	3x week for first 9 weeks; 4/5x week 15 weeks: 6 months	moderate	40%HRR-increase to upper moderate by week 9	Waitlist control	20min-30min	PSS	Aerobic exercise group sig. decrease in perceived stress (*p* < 0.05)
King et al.	Aerobic choice (walking/jogging)	3x week high intensity 5x week low intensity: 1 year	Moderate to hard	Low: 60-73% MHR High: 73-88% MHR after first 6 weeks	Medical and physical assessment only	Low: 30min High: 1 h	PSS	Sig. decrease for all conditions vs. control (*p* < 0.008), home-based low intensity + high intensity sig. decrease vs. group high intensity + control (*p* < 0.003)
Cormie et al.	Aerobic + Anaerobic (weights)	2x week: 3 months	Moderate//hard	Aerobic: 70-85% MHR				
Anaerobic: 6/12 MWR 1–4 sets (progressive and tailored)	Usual care	1 h	Distress (BSI-18)	Sig. decrease in psychological distress for exercise group (*p* = 0.045) vs. control				
Pourhabib et al.	Aerobic + Anaerobic (weights)	3x week: 3 months	light	Aerobic: slow walking Anaerobic: last 8 weeks: 500 g dumbbells	Heart Failure Education Program	1 h: 30 min walk, 30 min dumbbells	Psychological distress (MHI)	Sig. decrease psychological stress for exercise group (*p* < 0.001) vs. control
Imayama et al.	Aerobic + diet	5x week: 1 year	Moderate/hard	Gradual increase to 70-85% MHR sustained for final 10 months	no intervention	45 min	PSS	Aerobic exercise alone NS (*p* = 0.23), add diet education sig. = (*p* = 0.006)
Vaapio et al.	Anaerobic (lower-leg muscles)	1 year	NA	Individualised and progressive over trial	counselling	40-50 min:30 min + warmup and cooldown	Psychological distress (HR:QOL)	Sig. decrease stress for men (*p* = 0.029) and 65–74 age group (*p* = 0.037) vs. control

SEMCD-6: Self-efficacy for managing chronic disease, PSS: Perceived Stress Scale, NS: Not Significant, MHI: Mental Health Inventory, MHR: Max Heart Rate, MWR: Max Weight Repetition, HRR: Heart Rate Reserve, BPM: beats per minute

### 4.3. Exercise types

#### 4.3.1. Low-impact, low-intensity

Nearly half of the included studies investigated low-impact/low-intensity types of exercise such as qigong (Campo et al., 2014; Xiao et al., 2018; Zhang et al., 2016), yoga (Gothe et al., 2016), or dance (Ehlers et al., 2017). In this case, low-impact exercises are any movements that are easy on the joints and involve in-fluid motion. Qigong (‘qi’ = energy flow, ‘gong’ = achievement/skill) is an ancient Chinese martial art consisting of slow-flowing movements while practising deep breathing with a meditative focus to enhance ‘Qi’ or life energy to improve well-being (Rogers et al., 2009). All three studies using qigong showed statistical associations (*p* < 0.05 – implying decreases in stress post intervention compared to control) for separate populations: prostate cancer survivors, patients with COPD, and patients with chronic cardiovascular disease. Types of qigong utilised varied. Campo et al.’s intervention involved general qigong with a stretching control,, whilst Xiao et al.’s intervention involved Baduanjin qigong, a common form of qigong (Wang & Zhang, 2015) with a control involving usual activities. Their intervention includes 8 slow static movements. Zhang et al.’s intervention used Yijin Jing, which incorporates strength, force and flexibility, movement, and stillness, with mind and body working as one (Ding et al., 2014). Their control used usual care.

One study examined the effects of dancing on stress (Ehlers et al., 2017). They also included a walking only group and a walking and nutrition group. There were no differences between control and the dancing condition, though all conditions had significant decreases in stress, but not when compared to each other.

Yoga was the other type of intervention generally considered low impact. Gothe et al. (2016) investigated the effect of hatha yoga on stress but found no difference between yoga and their stretching controls. However, both the yoga group and control group showed significant decreases in stress from baseline and 8 weeks after.

#### 4.3.2. Aerobic exercise

Three studies investigated the effect of aerobic exercise (walking, jogging, or self-selected aerobic exercise) on stress. (Courneya et al., 2017; King et al., 1993; Puterman et al., 2018). Courneya et al. (2017), investigated self-selected aerobic exercise. They compared moderate volume to high volume exercise. They found no significant effect for perceived stress. Puterman et al. (2018) studied the effect of self-selected aerobic exercise. Only the exercise group had significant decreases in perceived stress. The third study (King et al., 1993) investigated individual walking/jogging at low intensity or high intensity and another group of supervised walking/jogging at high intensity. They found significant differences for all exercise groups combined vs the no-exercise control and significant differences between the two individual groups compared to group exercise and the control.

#### 4.3.3. Aerobic + Nutrition

One study (Imayama et al., 2011) looked at effects of moderate to vigorous aerobic exercise with and without nutritional education and its effect on overweight/obese older women’s’ stress. Aerobic exercise alone did not significantly alter perceived stress post-intervention, though in conjunction with diet education, it did decrease stress.

#### 4.3.4. Aerobic + Anaerobic

Two studies investigated the effect of aerobic exercise – a type of endurance exercise that uses oxygen – and added additional weight training (anaerobic – or without oxygen) (Cormie et al., 2015; Pourhabib et al., 2018). Cormie et al. (2015) researched the effect of moderate to high intensity aerobic exercise (bike, treadmill, rowing) and maximum repetitions on eight major muscle group machines. They found significant decreases in psychological distress after the intervention compared to controls. The other study included treadmill walking and resistance weights. They found significant decreases in stress compared to the control group.

#### 4.3.5. Anaerobic

The final study (Vaapio et al., 2007) investigated a weight training programme on older adults’ stress. Each exercise programme began with 5–10 min of warm up followed by exercises designed to improve lower leg muscle strength, balance, and coordination followed by a cool-down period of 5–10 min. The intensity of the exercises was planned separately for each participant and progressed according to their health status. They were performed sitting or standing depending on ability. The control group in this study received counselling education but no exercise. Results showed men in the active group had significant decreases in distress compared to controls. When divided into age brackets, the 65–74 age group showed significant decreases in distress compared to the age-matched controls.

### 4.4. Intensity

The intensity of each trial varied: one intervention did not have information concerning intensity (Vaapio et al., 2007). For the other eleven interventions, five used maximum heart rate (MHR), one used heart rate reserve (HRR) and one used Borg rating of perceived exertion. The other four did not explain how they measured intensity. Four interventions were described as light intensity (either 60% MHR) or an increase of less than 20 beats per minute (BPM), two were light/moderate (60–75% or 65% to 75% MHR), two were moderate (median of 4.3 on BORG scale and the other starting at 40% then increasing and maintaining an upper moderate zone of MHR), two were moderate/hard (70–85% MHR), and one had moderate (60–73% MHR) and hard conditions (73–88% MHR).

### 4.5. Duration

The length of the trials ranged between two months and a year. For the length of exercise, seven studies had one-hour interventions, one was 45 min, one was 40–50 min, one was 24 min, one was 35–40 min and increased to 65–70 min by the end of the trial, and one was 20 min and increased to 30 min by the end.

### 4.6. Participants’ health

Of the twelve trials, only four had healthy older adults. This is defined by investigators as not having any physical disease or serious conditions. They included family caregivers of people with dementia, healthy but low-active, and community dwelling adults with no cardiovascular disease/stroke. The other eight trials included: COPD, heart failure, history of falls, prostate cancer, high risk of breast cancer, cardiovascular disease, and overweight/obese.

### 4.7. Dropout

The dropout rate is important for exercise programmes for older adults. Research suggests high rates of dropout (20–50%) in the first three-six months of exercise trials (Dishman, 1991; Mullen et al., 2013; van der Deijl et al., 2014). King et al. (1993) did not include dropout rates or causes. The highest dropout rate was Campo et al. at 27.5%, while others ranged from 3.5% to 15.9%. Of the trials that included dropout rates, only three studies performed a dropout analysis in their results. Of those, one found differences in the dropout group versus the retained group. In separating by gender, they found that among males, dropouts were more likely to be living alone and have lower cognitive functioning. Among women, dropouts were more likely to be older and have lower cognitive, physical, and mental abilities. The relevant information gathered regarding dropout rates are in Table 5.

**Table 5.** Dropout analysis.

Author	Intervention dropout	Reasons	Control dropout	Control reasons	Attrition bias
Campo et al.	4	Health reasons (2), bad timing, no reason given	7	Health reasons (2), bad timing, too busy, not interested (2), family reasons	No differences between dropout group and retained group
Cormie et al.	1	Nauseous, Dizziness, fatigue (cancer therapy)	7	Desire to exercise (4), travel for assessment (2), time constraints	No dropout analysis performed
Courneya et al.	5	Medical reasons (2), nonadherent (2), personal reasons	9	Medical reasons (2), nonadherent, personal reasons (5), relocation	No dropout analysis performed
Ehlers et al.	Dance:12 Walk:6 Walk+:7	Reasons not given	14	Reasons not given	No dropout analysis performed
Gothe et al.	3	No longer interested, family emergency, time commitment	7	No longer interested (2), family emergency (2), time commitment, Health condition (sickness), travel	No dropout analysis performed
Imayama et al.	Exercise:11 exercise + Diet: 9	Exercise: Medical (2), transportation (2), work/family, death, other (5) Exercise + diet: work/family (2), medical, relocation, other (5)	7	Dissatisfied with randomisation (3), other reason (4)	No differences between dropout group and retained group
Pourhabib et al.	5	Missed programme sessions	2	No reason given	No dropout analysis performed
Puterman et al.	3	Medical, relocation, caregiver burden/stress	1	Dissatisfied with randomisation	No dropout analysis performed
Vaapio et al.	31	Death (5), Health (14), relocation, financial, study dissatisfaction (2), no reason (9)	29	Death (4), Health (9), financial, low motivation, study dissatisfaction (4), no reason (10)	Among men: higher cog. Function in retained vs dropout, dropouts more likely to be living alone Among women: retained: younger, better cognitive, physical, and mental abilities
Xiao et al.	1	Loss of contact	4	Loss of contact (4)	No dropout analysis performed
Zhang et al.	Qigong: 8 SME: 6	Qigong: Health (3), lost to follow-up (2), stopped exercise (3) SME: stopped exercise (4) health (2)	4	Loss of contact (4)	No dropout analysis performed

### 4.8. Risk of bias

For the RCTs in this review, most domains were evaluated as having low risk of bias. Three studies did not report or were unclear regarding their method of random sequence generation and three did not report or were unclear regarding allocation concealment. One did not use allocation concealment, presenting a high risk of bias. Two trials were assigned high risk in the performance bias category due to knowledge of the allocated interventions by participants while three were unclear. For detection bias, one was assigned high risk as they did not use blinding of the outcome assessment by research staff. Five trials did not mention assessment blinding. As with the majority of RCTs, selective reporting was unclear in all studies. Finally, when doing research on groups of older adults, health status may be an important contributor to how one deals with stress as well as the overall effect that exercise may have on them. Though a direct analysis was outside the scope of this review, in future reviews/research, it should be important to address this discrepancy.

## 5. Discussion

The prevalence of psychological stress amongst older adults necessitates the identification of a mechanism through which older populations can reduce stress. This review provides supporting evidence that exercise, particularly in the form of qigong, is associated with decreases in perceived stress. Aerobic exercise (walking, jogging, or biking) in tandem with anaerobic exercise (weightlifting), was also associated with reductions in stress. Aerobic training in tandem with diet education showed promising associations as well. Finally, strength training for men and young-old (65–74 years of age) participants provided some evidence for reduced stress. These results provide some consistency with regards to previous literature or systematic reviews on the role of specific exercise on stress management.

### 5.1. Mind–body exercise

The evidence in this review for mind–body exercises (yoga and qigong), provide relatively consistent results with previous research on younger healthier populations. One review, (Chong et al., 2011) which looked at yoga’s effect on stress showed positive results, though the authors emphasised there were methodological issues in almost all the trials included – short duration bouts of yoga, limited follow-up data, half the trials with no control, half did not have randomisation, and most had small samples. Also, they only included trials involving healthy adults between 18 and 65. Another more substantive review (Li & Goldsmith, 2012) examined 35 yoga trials focused on the effect of yoga in reducing anxiety and stress. The authors found the same methodological issues as the previous review.

One review (Wang et al., 2014) suggests that qigong, when practised between one and three months, is effective in reducing stress among healthy adults. Supporting evidence from the three studies on qigong may be due to the added meditation and breathing involved in this type of exercise that is not involved in other types. Although the yoga intervention did not reveal similar results for stress reduction could be explained by the more intense focus on physical positions, also called ‘asana’, rather than qigong’s focus on mind and breathing.

### 5.2. Multifactorial designs

The evidence for combining exercise and diet programmes is less clear for younger cohorts. One study (Kiernan et al., 2001) showed null results for stress reduction for overweight people when adding exercise to a diet programme. The belief is that community-dwelling overweight populations had such low levels of baseline stress that the exercise had little room to decrease stress. This stands in contrast to individuals who are overweight but in clinical or hospital-based diet programmes with much higher baseline stress (Wadden et al., 1997). Other suggestions for lack of change in stress were the degree of obesity in this study population, age (25–49), and type of exercise prescription (brisk walk or jogging at 60–80% MHR). Another study (Daubenmier et al., 2007) however, shows an interaction between reductions in dietary fat intake and an increase in exercise consumption in reducing stress. They believe the interaction of exercise, diet, and stress management are additive and interact in improving future outcomes. Although it is beyond the scope of this review, this combination of interventions shows great promise in being effective in reducing stress.

When combining aerobic and anaerobic exercise, there is not much consistent evidence. For reviews in the adult population, the most recent, (Elkington et al., 2017) looked at comparing aerobic, anaerobic, or combined trials and their effects on psychological well being (distress is a major factor). One trial combined both, one compared them, and two used only anaerobic exercise. The combined trial, (Maraki et al., 2005) had promising results, showing a twenty percent increase in psychological wellbeing. There were two other trials that examined the role of combined aerobic and anaerobic exercise programmes on perceived stress in the workplace. In the first trial, (Fischetti et al., 2019) police officers in the combined exercise intervention had significantly decreased levels of perceived stress after 8-weeks compared to the waitlist group. Another recent trial investigated perceived stress in the population of helping professions (police officers, doctors, psychologists, and teachers) (Greco, 2021). They found perceived stress decreased significantly in the combined exercise group while there were no changes in the waitlist group. Though these are encouraging results, it is only three small trials, one of which (Maraki et al., 2005) only included one bout of acute exercise rather than looking at the effects of a long-term programme.

When using anaerobic or strength training independent of other exercise types, the evidence is negative. In a previously mentioned review, (Elkington et al., 2017) only two trials investigated the use of resistance exercise alone in combating psychological distress. Of note, their review was only highlighting acute exercise (single dose) on psychological variables (pre-workout and post), limiting long term effects of any one type of exercise. One trial found no change between 5 min pre-exercise and immediately after. The other trial (Comstock et al., 2013) looked at psychological distress at baseline, immediately after, and 24 h after training. They found strength training increased psychological stress for people with lean body mass and that stress for those who were obese was even higher. This small amount of evidence runs counter to the study reviewed in this research. Reasons for this may be age or obesity level, but more likely the amount of weight lifted, repetitions, and that they only did the workout once rather than have their bodies adjust and test stress levels at the end of a longer trial. One possible explanation for Vaapio et al.’s positive results come from the multi-faceted fall-prevention programme where the exercise was in addition to a geriatric assessment and risk analysis, lectures, and social activities.

### 5.3. Trial length

The three trials that included qigong ran for 3, 4, and 6 months. The trial that ran for 6 months showed stronger effects the longer the trial was run for, providing some evidence that qigong trial duration in itself is important in reducing stress. Meanwhile, the yoga trial only ran for two months. While they showed an association between yoga and stress reduction, there were no statistical differences between the yoga group and the control. Perhaps, had it run longer, like that of the qigong trials, the effects would have been stronger. For the two multicomponent trials involving aerobic and anaerobic interventions, both used a three-month trial, and both had positive results, providing evidence that for combined exercise, three months is an adequate time period. For the aerobic and diet combined trial, and the anaerobic trial, both lasted for one year and had positive results, however, it is unknown if shorter trials would invoke the same results. For aerobic choice trials, the results were less clear. Two trials lasted a year, of which one had positive results while the other did not. The third, had a 6-month trial and had strong results for stress reduction. Finally, the dance trial lasted for six months but had no group differences for stress. The implications for these results suggest that the amount of time a study is conducted may provide further evidence of a linear relationship – longer study, stronger effect – in inducing stress buffering effects, especially as it relates to qigong.

### 5.4. Exercise duration

For the duration of each exercise, most used a one-hour intervention with mixed results: Four of seven had significant results while three did not. The other five trials included less than an hour for the exercise condition: two trials used roughly forty-five-minutes, and both had positive results. Two trials that used short duration exercise – twenty to thirty minutes – both showed positive associations. The final study started with 35–40 min and increased to 65–70 min, producing positive associations as well. These results provide varying degrees of support for trials that last for less than one hour and mixed results for exactly one hour. Thus, when conducting similar trials in the future, perhaps trials averaging between twenty to sixty minutes would be most effective. It also lends credence to the idea that older adults have enough attention to reduce stress by way of both short and long trials. That being said, it is important to note that no meta-analysis was conducted due to the heterogeneity of the trials. The relative importance of trial duration therefore cannot and should not be overstated and impossible to determine without more stringent analysis. This is also relevant for the following information regarding length of trial as well.

To understand optimal length of exercise in other populations, to help understand if/how older adults differ from the general public, it is necessary to investigate the literature. In one study, (Bhui & Fletcher, 2000), the exercise duration was important but only for between-gender differences (men had reduced amounts of stress the longer the exercise, while women had no significant differences in stress in relation to exercise duration). For trial length, a review conducted on exercise and stress (Sharon-David & Tenenbaum, 2017) found considerable variability in duration and no one length of time determined effectiveness. Two reviews specifically looked at exercise trial length in older adult populations. One (Theou et al., 2011) found there was not enough evidence to declare specific activity durations superior, though multicomponent trials lasting for five months or longer and three times per week, were most instrumental in producing positive health outcomes. Another review (Chase, 2013) found no difference in dosage, whether twelve weeks or a year.

## 6. Limitations

### 6.1. Generalizability

In this review, most trials were conducted on older adults with health concerns, e.g. cardiovascular disease, heart disease, cancer treatment, obesity, COPD, and others. Older adults with these conditions may have stress that does not generalise to healthy older adults.

### 6.2. Self-report exercise

Any trials using self-reported exercise interventions should be properly scrutinised given the disparity between perceived physical fitness and health when compared to objective measurements (Wells et al., 2016). Self-report physical activity has shown to be commonly overestimated (Lee et al., 2011; Prince et al., 2008). When attempting to understand which types were overestimated and by how much, both moderate and vigorous activity were overestimated by 42 and 39 min a day respectively, while sedentary time was underestimated by more than 2 h (Schaller et al., 2016). This is substantial when considering most trials in this review consisted of sixty-minutes of exercise. While limiting inclusion to studies implementing supervised exercise was done to reduce the effect of a positivity bias surrounding self-report exercise, it did eliminate effective interventions that may have used self-report exercise in an unbiased fashion.

### 6.3. High intensity

The studies included in this review involved predominantly older adults with health concerns. Thus, only one of the trials included a group who endured a high intensity intervention, while two involved moderate to hard intensities. This is most likely due to concerns from participants and researchers regarding safe exercise levels. Because of this, it is difficult to make comparisons regarding which intensity is optimal for stress management.

### 6.4. Bias

With regards to exercise interventions, there is a risk of a publication bias. It is possible only trials involving positive stress-reducing results are published. This risk is especially important with systematic reviews with a low number of studies (Ioannidis, 2005). Although this review included trials with negative results for decreases in stress, often stress was a secondary outcome.

### 6.5. Motivation

Although all included studies involved RCTs and supervised exercise, exercise motivation remains an issue for older adults. In real-world scenarios, especially in North America, older adults make up a larger proportion of sedentary populations (Diaz et al., 2016; Prince et al., 2020). It is not enough to state qigong or aerobic exercise with nutrition education or aerobic and weight training are the most beneficial and that increased duration is better for reducing stress. While knowledge is always the first step towards progress of understanding, if exercise uptake is insufficient, in other words, knowledge without action, possible stress reduction and improvements in quality of life will likely not occur.

### 6.6. Age cut-off

With regards to the inclusion criteria using an age restriction (50 years of age or older), this may have ultimately limited the breadth of the review. There is not a perfect cut-off with respect to defining older adults. However, in using 50+, the review was able to include many more trials whereas using 65+, which many consider as defining of old age, there would only have been two trials involved. Another point of contention with regards to age is the issue regarding age limits at all, especially when considering the marked differences between the physical abilities of someone at 50 versus somebody at 65 or 80. The 50 + cut-off was implemented to cast a wider net of eligible studies especially considering the lower potential for exercise trials involving participants over 65.

### 6.7. Exercise variability

This systematic review, perhaps due to its subject matter, or perhaps due to lack of variability in most exercise programmes, is limited in scope due to ten of the twelve studies dealing with either general aerobic exercise or qigong. Because of these limitations, it is somewhat difficult to say for certain which exercise is best and for whom for reducing psychological stress.

## 7. Future directions/conclusions

To our knowledge, this is the first review to synthesise information on efficacy of exercise interventions in reducing psychological stress in adults aged 50 + . Knowing the optimal type, intensity, and duration of exercise in combating stress may improve tailoring and targeting strategies among health practitioners and/or family caregivers and older adults themselves. It would help direct recreational aides to better navigate best practices and to help motivate their clients in choosing the exercise right for them. For stress reduction, intensity of exercise may not be the only important element, since there is also evidence that mindfulness and medication may also be relevant.

One major takeaway of this review is that the methods of activity that are most effective in reducing stress are affordable and can be done within the confines of older adults’ homes or neighbourhoods. This is of increasing importance because affordability and lack of time are considered two of the most cited barriers for why adults remain sedentary (Reichert et al., 2007).

Exercise interventions are advantageous in that they are accessible, can be performed with little setup, and most populations can participate either individually or in groups. Once these interventions are developed, adequately tested, and tailored to individual needs of older adults, they can become a primary mechanism for stress management. Mounting evidence shows exercise is effective in reducing stress and associated symptoms both independently, combined with other strategies and when directly compared to other techniques such as mindfulness (Pinniger et al., 2012).

For future research in this area, a better understanding of motivation and adherence would help provide additional management skills for a stress reduction toolbox for older adults as they negotiate the ongoing pandemic and other stressors. As well, research identifying which groups of older adults (those with cancer vs cardiovascular disease vs COPD vs frailty) benefit from which types of exercise would be useful. The results from this review provide support for emphasising stress management in government physical activity guidelines targeting adults over the age of 50.

## References

[CIT0001] American Psychological Association. (2022a). What’s the difference between stress and anxiety? Retrieved March 26, 2022, from https://www.apa.org/topics/stress-anxiety-difference

[CIT0002] American Psychological Association. (2022b). Depression. *In APA dictionary of psychology.* Retrieved March 26, 2022, from https://dictionary.apa.org/depression

[CIT0003] Bhui, K., & Fletcher, A. (2000). Common mood and anxiety states: Gender differences in the protective effect of physical activity. *Social Psychiatry and Psychiatric Epidemiology*, *35*(1), 28–35. 10.1007/s001270050005 10741533

[CIT0004] Campo, R. A., Agarwal, N., LaStayo, P. C., O’Connor, K., Pappas, L., Boucher, K. M., Gardner, J., Smith, S., Light, K. C., & Kinney, A. Y. (2014). Levels of fatigue and distress in senior prostate cancer survivors enrolled in a 12-week randomized controlled trial of Qigong. *Journal of Cancer Survivorship*, *8*(1), 60–69. 10.1007/s11764-013-0315-5 24170679 PMC3945387

[CIT0005] Centers for Disease Control and Prevention. (2017). Glossary of terms. Retrieved March 23, 2022, from https://www.cdc.gov/nchs/nhis/physical_activity/pa_glossary.htm

[CIT0006] Chase, J. A. D. (2013). Physical activity interventions among older adults: A literature review. *Research and Theory for Nursing Practice*, *27*(1), 53–80. 10.1891/1541-6577.27.1.53 23923347 PMC4523118

[CIT0007] Chong, C. S., Tsunaka, M., & Chan, E. P. (2011). Effects of yoga on stress management in healthy adults: A systematic review. *Alternative Therapies in Health and Medicine*, *17*(1), 32–38. 21614942

[CIT0008] Chrousos, G. P., & Gold, P. W. (1992). The concepts of stress and stress system disorders: Overview of physical and behavioral homeostasis. *Jama*, *267*(9), 1244–1252. 10.1001/jama.1992.03480090092034 1538563

[CIT0009] Cohen, S., Kamarck, T., & Mermelstein, R. (1983). A global measure of perceived stress. *Journal of Health and Social Behavior*, *24*(4), 385–396. 10.2307/2136404 6668417

[CIT0010] Comstock, B. A., Thomas, G. A., Dunn-Lewis, C., Volek, J. S., Szivak, T. K., Hooper, D. R., Kupchak, B. R., Flanagan, S. D., Denegar, C. R., & Kraemer, W. J. (2013). Effects of acute resistance exercise on muscle damage and perceptual measures between men who are lean and obese. *The Journal of Strength & Conditioning Research*, *27*(12), 3488–3494. 10.1519/JSC.0b013e31828f8202 23478480

[CIT0011] Cormie, P., Galvão, D. A., Spry, N., Joseph, D., Chee, R., Taaffe, D. R., Chambers, S. K., & Newton, R. U. (2015). Can supervised exercise prevent treatment toxicity in patients with prostate cancer initiating androgen-deprivation therapy: A randomised controlled trial. *BJU International*, *115*(2), 256–266. 10.1111/bju.12646 24467669

[CIT0012] Courneya, K. S., McNeil, J., O'Reilly, R., Morielli, A. R., & Friedenreich, C. M. (2017). Dose-response effects of aerobic exercise on quality of life in postmenopausal women: Results from the breast cancer and exercise trial in alberta (BETA). *Annals of Behavioral Medicine*, *51*(3), 356–364. 10.1007/s12160-016-9859-8 27837524

[CIT0013] Daubenmier, J. J., Weidner, G., Sumner, M. D., Mendell, N., Merritt-Worden, T., Studley, J., & Ornish, D. (2007). The contribution of changes in diet, exercise, and stress management to changes in coronary risk in women and men in the multisite cardiac lifestyle intervention program. *Annals of Behavioral Medicine*, *33*(1), 57–68. 10.1207/s15324796abm3301_7 17291171

[CIT0014] Diaz, K. M., Howard, V. J., Hutto, B., Colabianchi, N., Vena, J. E., Blair, S. N., & Hooker, S. P. (2016). Patterns of sedentary behavior in US middle-age and older adults: The REGARDS study. *Medicine and Science in Sports and Exercise*, *48*(3), 430–438. 10.1249/MSS.0000000000000792 26460633 PMC4760895

[CIT0015] Ding, M., Zhang, W., Li, K., & Chen, X. (2014). Effectiveness of t'ai chi and qigong on chronic obstructive pulmonary disease: A systematic review and meta-analysis. *The Journal of Alternative and Complementary Medicine*, *20*(2), 79–86. 10.1089/acm.2013.0087 23961940 PMC3924809

[CIT0016] Dishman, R. K. (1991). Increasing and maintaining exercise and physical activity. *Behavior Therapy*, *22*(3), 345–378. 10.1016/S0005-7894(05)80371-5

[CIT0017] Ehlers, D. K., Daugherty, A. M., Burzynska, A. Z., Fanning, J., Awick, E. A., Chaddock-Heyman, L., Kramer, A. F., & McAuley, E. (2017). Regional brain volumes moderate, but do not mediate, the effects of group-based exercise training on reductions in loneliness in older adults. *Frontiers in Aging Neuroscience*, *9*, 110. 10.3389/fnagi.2017.00110 28487648 PMC5403947

[CIT0018] Elkington, T. J., Cassar, S., Nelson, A. R., & Levinger, I. (2017). Psychological responses to acute aerobic, resistance, or combined exercise in healthy and overweight individuals: A systematic review. *Clinical Medicine Insights: Cardiology*, *11*, 1–23. 10.1177/1179546817701725 PMC5404906 28469495

[CIT0019] Fischetti, F., Cataldi, S., Latino, F., & Greco, G. (2019, June 14-15). *Effectiveness of multilateral training didactic method on physical and mental wellbeing in law enforcement* [Conference presentation]. Spring Conferences of Sports Science: Costa Blanca Sports Science Events, Alicante, Spain.

[CIT0020] Forsman, A. K., Schierenbeck, I., & Wahlbeck, K. (2011). Psychosocial interventions for the prevention of depression in older adults: Systematic review and meta-analysis. *Journal of Aging and Health*, *23*(3), 387–416. 10.1177/0898264310378041 20935250

[CIT0021] Gerber, M., Holsboer-Trachsler, E., Pühse, U., & Brand, S. (2016). Exercise is medicine for patients with major depressive disorders: But only if the “pill” is taken!. *Neuropsychiatric Disease and Treatment*, *12*, 1977–1981. 10.2147/NDT.S110656 27540294 PMC4981216

[CIT0022] Gerber, M., & Pühse, U. (2009). Do exercise and fitness protect against stress-induced health complaints? A review of the literature. *Scandinavian Journal of Public Health*, *37*(8), 801–819. 10.1177/1403494809350522 19828772

[CIT0023] Gothe, N. P., Keswani, R. K., & McAuley, E. (2016). Yoga practice improves executive function by attenuating stress levels. *Biological Psychology*, *121*, 109–116. 10.1016/j.biopsycho.2016.10.010 27794449

[CIT0024] Greco, G. (2021). Effects of combined exercise training on work-related burnout symptoms and psychological stress in the helping professionals. *Journal of Human Sport and Exercise*, *16*(2), 424–434. 10.14198/jhse.2021.162.16

[CIT0025] Imayama, I., Alfano, C. M., Kong, A., Foster-Schubert, K. E., Bain, C. E., Xiao, L., Duggan, C., Wang, C.-Y., Campbell, K. L., Blackburn, G. L., & McTiernan, A. (2011). Dietary weight loss and exercise interventions effects on quality of life in overweight/obese postmenopausal women: A randomized controlled trial. *International Journal of Behavioral Nutrition and Physical Activity*, *8*(1), 1–12. 10.1186/1479-5868-8-118 22026966 PMC3215656

[CIT0026] Ioannidis, J. P. (2005). Why most published research findings are false. *PLoS Medicine*, *2*(8), e124. 10.1371/journal.pmed.0020124 16060722 PMC1182327

[CIT0027] Jørgensen, L., Paludan-Müller, A. S., Laursen, D. R., Savović, J., Boutron, I., Sterne, J. A., Higgins, J. P. T., & Hróbjartsson, A. (2016). Evaluation of the Cochrane tool for assessing risk of bias in randomized clinical trials: overview of published comments and analysis of user practice in Cochrane and non-Cochrane reviews. *Systematic Reviews*, *5*(1), 1–13. 10.1186/s13643-016-0259-8 27160280 PMC4862216

[CIT0028] Juster, R. P., McEwen, B. S., & Lupien, S. J. (2010). Allostatic load biomarkers of chronic stress and impact on health and cognition. *Neuroscience & Biobehavioral Reviews*, *35*(1), 2–16. 10.1016/j.neubiorev.2009.10.002 19822172

[CIT0029] Kiernan, M., King, A. C., Stefanick, M. L., & Killen, J. D. (2001). Men gain additional psychological benefits by adding exercise to a weight-loss program. *Obesity Research*, *9*(12), 770–777. 10.1038/oby.2001.106 11743061

[CIT0030] King, A. C., Taylor, C. B., & Haskell, W. L. (1993). Effects of differing intensities and formats of 12 months of exercise training on psychological outcomes in older adults. *Health Psychology*, *12*(4), 292–300. 10.1037/0278-6133.12.4.292 8404803

[CIT0031] Lazarus, R. S., & Folkman, S. (1984). *Stress, appraisal, and coping*. Springer publishing company.

[CIT0032] Lee, P. H., Macfarlane, D. J., Lam, T. H., & Stewart, S. M. (2011). Validity of the international physical activity questionnaire short form (IPAQ-SF): A systematic review. *International Journal of Behavioral Nutrition and Physical Activity*, *8*(1), 1–11. 10.1186/1479-5868-8-1 22018588 PMC3214824

[CIT0033] Li, A. W., & Goldsmith, C. A. W. (2012). The effects of yoga on anxiety and stress. *Alternative Medicine Review*, *17*, 21–35. 22502620

[CIT0034] Maraki, M., Tsofliou, F., Pitsiladis, Y. P., Malkova, D., Mutrie, N., & Higgins, S. (2005). Acute effects of a single exercise class on appetite, energy intake and mood. Is there a time-of-day effect? *. Appetite*, *45*(3), 272–278. 10.1016/j.appet.2005.07.005 16157416

[CIT0035] Marengoni, A., Angleman, S., Melis, R., Mangialasche, F., Karp, A., Garmen, A., Meinow, B., & Fratiglioni, L. (2011). Aging with multimorbidity: A systematic review of the literature. *Ageing Research Reviews*, *10*(4), 430–439. 10.1016/j.arr.2011.03.003 21402176

[CIT0036] Mochcovitch, M. D., Deslandes, A. C., Freire, R. C., Garcia, R. F., & Nardi, A. E. (2016). The effects of regular physical activity on anxiety symptoms in healthy older adults: A systematic review. *Brazilian Journal of Psychiatry*, *38*(3), 255–261. 10.1590/1516-4446-2015-1893 27579597 PMC7194273

[CIT0037] Moher, D., Shamseer, L., Clarke, M., Ghersi, D., Liberati, A., Petticrew, M., Shekelle, P., & Stewart, L. A. (2015). Preferred reporting items for systematic review and meta-analysis protocols (PRISMA-P) 2015 statement. *Systematic Reviews*, *4*(1), 1–9. 10.1186/2046-4053-4-1 25554246 PMC4320440

[CIT0038] Mullen, S. P., Wójcicki, T. R., Mailey, E. L., Szabo, A. N., Gothe, N. P., Olson, E. A., Fanning, J., Kramer, A., & McAuley, E. (2013). A profile for predicting attrition from exercise in older adults. *Prevention Science*, *14*(5), 489–496. 10.1007/s11121-012-0325-y 23412942 PMC3806454

[CIT0039] Nielsen, N. R., Kristensen, T. S., Schnohr, P., & Grønbæk, M. (2008). Perceived stress and cause-specific mortality among men and women: Results from a prospective cohort study. *American Journal of Epidemiology*, *168*(5), 481–491. 10.1093/aje/kwn157 18611955

[CIT0040] Pinniger, R., Brown, R. F., Thorsteinsson, E. B., & McKinley, P. (2012). Argentine tango dance compared to mindfulness meditation and a waiting-list control: A randomised trial for treating depression. *Complementary Therapies in Medicine*, *20*(6), 377–384. 10.1016/j.ctim.2012.07.003 23131367

[CIT0041] Popay, J., Roberts, H., Sowden, A., Petticrew, M., Arai, L., Rodgers, M., … Duffy, S. (2006). Guidance on the conduct of narrative synthesis in systematic reviews. *A product from the ESRC methods programme, Version*, *1*, b92.

[CIT0042] Pourhabib, A., Fotokian, Z., Nasiri, M., & Abrotan, S. (2018). Effects of a group-based aerobic and resistance exercise program on physiological-psychological adaptation in elderly with heart failure. *Journal of Clinical Gerontology and Geriatrics*, *9*(2), 59–66. 10.24816/jcgg.2018.v9i2.05

[CIT0043] Prince, S. A., Adamo, K. B., Hamel, M. E., Hardt, J., Gorber, S. C., & Tremblay, M. (2008). A comparison of direct versus self-report measures for assessing physical activity in adults: A systematic review. *International Journal of Behavioral Nutrition and Physical Activity*, *5*(1), 1–24. 10.1186/1479-5868-5-56 18990237 PMC2588639

[CIT0044] Prince, S. A., Melvin, A., Roberts, K. C., Butler, G. P., & Thompson, W. (2020). Sedentary behaviour surveillance in Canada: Trends, challenges and lessons learned. *International Journal of Behavioral Nutrition and Physical Activity*, *17*(1), 1–21. 10.1186/s12966-019-0902-6 32151285 PMC7063715

[CIT0045] Puterman, E., Weiss, J., Lin, J., Schilf, S., Slusher, A. L., Johansen, K. L., & Epel, E. S. (2018). Aerobic exercise lengthens telomeres and reduces stress in family caregivers: A randomized controlled trial-Curt Richter award paper 2018. *Psychoneuroendocrinology*, *98*, 245–252. 10.1016/j.psyneuen.2018.08.002 30266522

[CIT0046] Reichert, F. F., Barros, A. J., Domingues, M. R., & Hallal, P. C. (2007). The role of perceived personal barriers to engagement in leisure-time physical activity. *American Journal of Public Health*, *97*(3), 515–519. 10.2105/AJPH.2005.070144 17267731 PMC1805028

[CIT0047] Rogers, C. E., Larkey, L. K., & Keller, C. (2009). A review of clinical trials of tai chi and qigong in older adults. *Western Journal of Nursing Research*, *31*(2), 245–279. 10.1177/0193945908327529 19179544 PMC2810462

[CIT0048] Schaller, A., Rudolf, K., Dejonghe, L., Grieben, C., & Froboese, I. (2016). Influencing factors on the overestimation of self-reported physical activity: A cross-sectional analysis of low back pain patients and healthy controls. *BioMed Research International*, *2016*, 1497213. 10.1155/2016/1497213 27298820 PMC4889825

[CIT0049] Scott, S. B., Jackson, B. R., & Bergeman, C. S. (2011). What contributes to perceived stress in later life? A recursive partitioning approach. *Psychology and Aging*, *26*(4), 830–843. 10.1037/a0023180 21604885 PMC3177031

[CIT0050] Sharon-David, H., & Tenenbaum, G. (2017). The effectiveness of exercise interventions on coping with stress: Research synthesis. *Studies in Sport Humanities*, *22*(22), 19–29. 10.5604/01.3001.0012.6520

[CIT0051] Sterne, J. A., Savović, J., Page, M. J., Elbers, R. G., Blencowe, N. S., Boutron, I., … Higgins, J. P. (2019). Rob 2: A revised tool for assessing risk of bias in randomised trials. *BMJ*, *366*. 10.1136/bmj.l4898 31462531

[CIT0052] Theou, O., Stathokostas, L., Roland, K. P., Jakobi, J. M., Patterson, C., Vandervoort, A. A., & Jones, G. R. (2011). The effectiveness of exercise interventions for the management of frailty: A systematic review. *Journal of Aging Research*, *2011*, 1–19. 10.4061/2011/569194 PMC3092602 21584244

[CIT0053] Unger, J. B., Johnson, C. A., & Marks, G. (1997). Functional decline in the elderly: Evidence for direct and stress-buffering protective effects of social interactions and physical activity. *Annals of Behavioral Medicine*, *19*(2), 152–160. 10.1007/BF02883332 9603690

[CIT0054] Vaapio, S., Salminen, M., Vahlberg, T., Sjösten, N., Isoaho, R., Aarnio, P., & Kivelä, S. L. (2007). Effects of risk-based multifactorial fall prevention on health-related quality of life among the community-dwelling aged: A randomized controlled trial. *Health and Quality of Life Outcomes*, *5*(1), 1–8. 10.1186/1477-7525-5-20 17462083 PMC1868017

[CIT0055] van der Deijl, M., Etman, A., Kamphuis, C. B., & van Lenthe, F. J. (2014). Participation levels of physical activity programs for community-dwelling older adults: A systematic review. *BMC Public Health*, *14*(1), 1–8. 10.1186/1471-2458-14-1301 25523712 PMC4301079

[CIT0056] Wadden, T. A., Vogt, R. A., Andersen, R. E., Bartlett, S. J., Foster, G. D., Kuehnel, R. H., Wilk, J., Weinstock, R., Buckenmeyer, P., Berkowitz, R. I., & Steen, S. N. (1997). Exercise in the treatment of obesity: Effects of four interventions on body composition, resting energy expenditure, appetite, and mood. *Journal of Consulting and Clinical Psychology*, *65*(2), 269. 10.1037/0022-006X.65.2.269 9086690

[CIT0057] Wang, C. W., Chan, C. H., Ho, R. T., Chan, J. S., Ng, S. M., & Chan, C. L. (2014). Managing stress and anxiety through qigong exercise in healthy adults: A systematic review and meta-analysis of randomized controlled trials. *BMC Complementary and Alternative Medicine*, *14*(1), 1–9. 10.1186/1472-6882-14-1 24400778 PMC3893407

[CIT0058] Wang, C. Y., & Zhang, H. (2015). Influence of Baduanjin combined with routine treatment on blood glucose level in type 2 diabetic patients. *China Medicine and Pharmacy*, *5*(22), 49–52.

[CIT0059] Wayne, P. M., Walsh, J. N., Taylor-Piliae, R. E., Wells, R. E., Papp, K. V., Donovan, N. J., & Yeh, G. Y. (2014). Effect of Tai Chi on cognitive performance in older adults: Systematic review and meta-analysis. *Journal of the American Geriatrics Society*, *62*(1), 25–39. 10.1111/jgs.12611 24383523 PMC4055508

[CIT0060] Wells, E. K., Avery, M. L., Eschbach, L. C., & Bunn, J. (2016). A comparison of perceived physical fitness and objective measurements. *Sport Journal*, 1–1.

[CIT0061] Xiao, X., Wang, J., Gu, Y., Cai, Y., & Ma, L. (2018). Effect of community-based practice of Baduanjin on self-efficacy of adults with cardiovascular diseases. *PloS One*, *13*(7), e0200246. 10.1371/journal.pone.0200246 30059552 PMC6066212

[CIT0062] Zhang, M., Xv, G., Luo, C., Meng, D., & Ji, Y. (2016). Qigong Yi Jinjing promotes pulmonary function, physical activity, quality of life and emotion regulation self-efficacy in patients with chronic obstructive pulmonary disease: A pilot study. *The Journal of Alternative and Complementary Medicine*, *22*(10), 810–817. 10.1089/acm.2015.0224 27487437

